# Artificial Intelligence in Nursing Decision-Making: A Bibliometric Analysis of Trends and Impacts

**DOI:** 10.3390/nursrep15060198

**Published:** 2025-06-03

**Authors:** Mengdie Hu, Yan Wang, Yunsong Liu, Bingqing Cai, Fanjing Kong, Qian Zheng, Dan Zhao, Guanghui Gao, Zhouguang Hui

**Affiliations:** 1 Department of VIP Medical Services, National Cancer Center/National Clinical Research Center for Cancer/Cancer Hospital, Chinese Academy of Medical Sciences and Peking Union Medical College, Beijing 100021, China; hmd228417854@126.com (M.H.);; 2 China Department of Radiation Oncology, National Cancer Center/National Clinical Research Center for Cancer/Cancer Hospital, Chinese Academy of Medical Sciences and Peking Union Medical College, Beijing 100021, China

**Keywords:** artificial intelligence, nursing, decision making, bibliometrics

## Abstract

**Background:** Nursing decision-making is pivotal for patient safety and care quality. While artificial intelligence (AI) offers transformative potential in this field, a comprehensive analysis of global research trends is lacking. **Methods:** We conducted a bibliometric analysis of 238 publications (197 research papers, 41 reviews) from the Web of Science Core Collection (2003–2025) using CiteSpace and VOSviewer. **Results:** The results reveal growing interest (7.59% annually) in the field of AI in nursing decision-making, with contributions from 54 countries/regions. The USA leads in the number of publications, followed by China and Canada, while the United Kingdom stands out in terms of citation impact. Institutions such as Columbia University and Harvard Medical School dominate in both the publication volume and citation frequency. Journal analysis shows that the top three journals in terms of publication volume in this field are *Cin-Computers Informatics Nursing*, *Journal of Nursing Management*, and *Applied Clinical Informatics*. Keyword analysis highlights the significant potential of natural language processing technologies, particularly those based on large language models (e.g., ChatGPT), in nursing decision-making. Furthermore, emerging trends are evident, with the sudden appearance and rapid growth of keywords such as “patient safety” and “user acceptance”, indicating a shift in research focus from purely technology-driven studies to a greater emphasis on the practical impact of AI technologies on nursing systems and their clinical applications. **Conclusions:** This study delineates the current landscape and evolving trends of AI in nursing decision-making, emphasizing its progression from theoretical frameworks to clinical integration, thereby providing valuable references for future research.

## 1. Introduction

Nursing decision-making is at the core of healthcare, exerting a direct influence on patient outcomes and the quality of care [1,2]. Traditional nursing decision-making primarily relies on nurses’ experience and intuition. With the widespread adoption of evidence-based practice, nursing has progressively integrated scientific research, clinical guidelines, and patient data to promote standardized and scientific decision-making [3,4]. However, with the exponential growth of medical data and the increasing complexity of patient needs, this model faces significant challenges [5]. The rapid development of artificial intelligence (AI) has brought new opportunities to nursing decision-making. AI technologies, such as machine learning and natural language processing, can analyze vast amounts of data, identify complex patterns, and provide data-driven decision support, thereby assisting nurses in making more accurate and efficient decisions [6,7,8].

In recent years, research on the application of AI in nursing has experienced exponential growth, covering multiple dimensions such as clinical diagnostic support, patient risk assessment, and optimization of care pathways [9,10,11]. For example, Martinez, Juan Manuel et al. developed a system for predicting the probability of emergency department admissions based on logistic regression and artificial neural networks [12]. This system achieved high-precision predictions using routine triage data, enabling nursing staff to allocate resources in advance. In addition, Reilly, Christian et al. developed a predictive model for emergency severity indices by clinical natural language processing and machine-learning algorithms (KATE) [13]. The research results demonstrated that KATE’s accuracy in assigning triage severity levels significantly outperformed that of nurses. On the contrary, Ilana, Dubovi et al. have evaluated the clinical decision-making abilities of registered nurses, nursing students, and ChatGPT. The results indicate that generative AI tools exhibit tendencies toward indecisiveness and overclassification [14]. These contrasting outcomes highlight the complexity and variability in AI’s role within nursing practice. Therefore, it is necessary to conduct a systematic literature analysis of the existing literature to elucidate the current research status and developmental trajectories.

While research on AI in nursing decision-making has expanded rapidly, a systematic mapping of global trends in this field, including publication patterns, dominant themes, and research efforts, remains lacking. Such an analysis would help chart the field’s development, identify key information sources, evaluate the true impact of journals and publications, and uncover emerging research directions. Although several systematic reviews have addressed AI applications in nursing, these primarily focus on technical validation and clinical implementation challenges. These review methodologies fail to achieve our objective of a comprehensive literature analysis focused specifically on AI technologies in nursing decision-making. Bibliometric analysis, a quantitative approach employing statistical methods to evaluate the scientific literature, serves as a powerful tool for visualizing research progress and identifying trends in specialized fields [15]. Currently, bibliometrics has gained widespread application in nursing research [16,17,18,19,20,21]. For example, there have been bibliometric analyses exploring research trends in AI in elderly care, as well as research hotspots and thematic trends in nursing education [22,23]. Yet despite growing interest, no bibliometric study has systematically assessed the global trends and emerging areas in AI for nursing decision-making. Thus, a systematic bibliometric analysis of AI in the nursing decision-making literature is essential to establish a foundational reference for future research.

Therefore, in this study, we conducted a bibliometric analysis of papers related to “AI in nursing decision-making”. Using data extracted from the Web of Science Core Collection database, we systematically analyzed the publication trends, identified the most influential countries, institutions, and researchers, identified research hotspots, and explored future research directions. This study aims to systematically reveal the research trends and knowledge evolution of AI in nursing decision-making through bibliometric methods. By analyzing research output metrics and network visualizations, it evaluates the academic influence of individuals, institutions, and countries, while uncovering the spatial distribution characteristics of knowledge production and transnational academic communities. Through co-citation clustering and keyword timeline analysis, it identifies the transformative effects of core technologies, such as machine learning and natural language processing, on nursing decision-making research paradigms, as well as key research themes and emerging trends. Our analysis provides a better understanding of the current status and potential of AI in nursing decision-making and provides a valuable resource for researchers.

## 2. Materials and Methods

### 2.1. Data Collection

**Figure 1:**
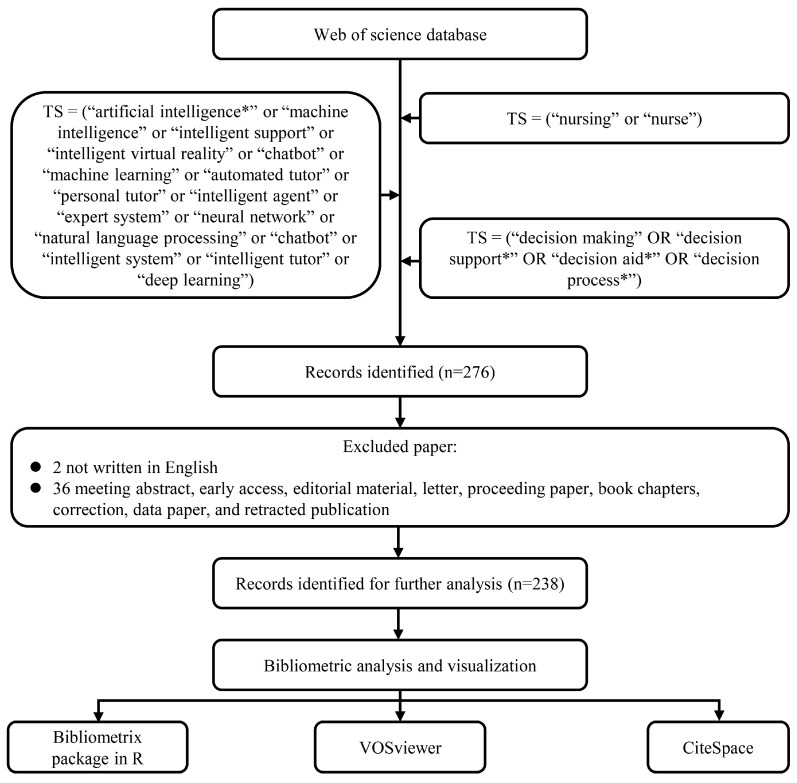
Flowchart of the search strategy and exclusion criteria. The * denotes a wildcard to capture variant word endings.

We systematically searched the Web of Science Core Collection (WOSCC) on 15 January 2025, using the following query strategy: TS = (“artificial intelligence*” or “machine intelligence” or “intelligent support” or “intelligent virtual reality” or “chatbot” or “machine learning” or “automated tutor” or “personal tutor” or “intelligent agent” or “expert system” or “neural network” or “natural language processing” or “chatbot” or “intelligent system” or “intelligent tutor” or “deep learning”) AND TS = (“nursing” or “nurse”) AND TS = (“decision making” OR “decision support*” OR “decision aid*” OR “decision process*”). This project focuses on published papers and reviews, as these document types are typically peer-reviewed, ensuring the quality and applicability of the bibliometric analysis. Only papers and reviews published in English were included. A total of 238 records were selected for analysis. The specific literature-screening process is illustrated in Figure 1. This search strategy was developed in collaboration with Fanjing Kong, a senior librarian from Peking Union Medical College.

### 2.2. Data Analysis

Bibliometric analysis, a quantitative approach that uses statistical methods to evaluate scientific publications, provides a powerful tool for visualizing scientific progress and identifying emerging trends within a specific research field [15]. This methodology allows for the systematic analysis of publication patterns, citation networks, and cooccurring keywords, offering an objective measure of scientific output and impact. We used the literature records from Section 2.1 as input files and performed statistical analyses using the following methods.

CiteSpace (version = 6.4.R1) [24] was used for a dualmap overlay analysis of journals, keyword clustering, timeline views of keywords, and explosive keywords. In the clustering analysis generated by CiteSpace software, the modularity Q value (Q) and weighted mean silhouette S value (S) are key metrics for evaluating the robustness of network clustering. The Q value measures the tightness of the connections between nodes assigned to the same cluster within a network, with a range of [−1, 1]. A Q value closer to 1 indicates stronger internal connections within clusters and weaker inter-cluster connections, signifying better clustering performance. Typically, Q > 0.3 is considered indicative of a significant cluster structure. The S value assesses the degree to which individual nodes match their assigned clusters, ranging from [−1, 1]. An S > 0.5 suggests high intra-cluster consistency, while S > 0.7 indicates highly reliable clustering results.

VOSviewer (version = 1.6.20) [25] was employed for statistics on publication volume by country, inter-country collaboration networks, publication volume by institution, inter-institution collaboration networks, publication volume by author, and inter-author collaboration networks. The bibliometrix package (version = 4.3.0) in R was used for annual publication volume statistics, total citations by country, and average citations per country.

### 2.3. Data Processing

We manually merged synonyms. We combined synonyms for countries, such as replacing “Peoples R China” and “Taiwan” with “China”; we merged synonyms for institutions, such as replacing “chinese acad med sci & peking union med coll” with “chinese acad med sci”; and we merged synonyms for keywords, such as replacing “artificial intelligence” with “ai”.

## 3. Results

### 3.1. Overview of Publication Status

**Figure 2:**
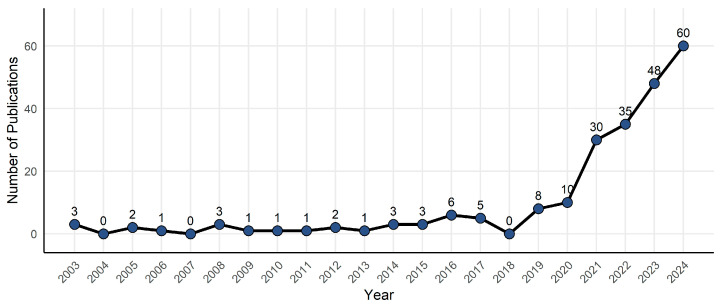
Number of papers published from 2003 to 2024.

A total of 238 publications were retrieved from the Web of Science Core Collection database, including 197 research papers (82.77%) and 41 review papers (17.23%). Using the bibliographic records exported from the database as input files, we performed statistical analyses on annual publication counts, authors, and countries/regions using R’s bibliometrix package. The results showed that the average citations per publication was 15.58. Researchers from 54 countries/regions and 1394 authors have contributed to the research on AI in nursing decision-making, with their findings published in 145 journals. Figure 2 shows a significant increasing trend in the number of publications related to AI in nursing decision-making, demonstrating a 7.59% compound annual growth rate. Based on the annual number of published papers, the research can be divided into two stages. Phase I (2003–2018) was the initial exploration stage, with fewer than 10 papers published each year. Phase II (2019–2024) saw an exponential growth in the annual number of published papers, as more and more scholars applied AI to nursing decision-making. We can expect that the research development in this field will not stop accelerating in the future.

### 3.2. Contribution of Countries to Publications

**Figure 3:**
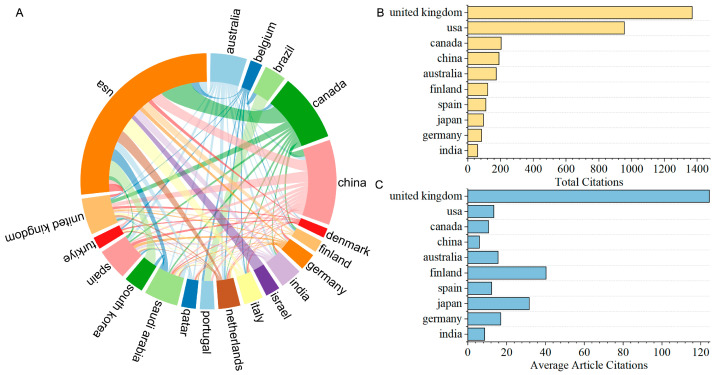
Contribution of country/region to publications. (**A**) The top 20 countries/regions by publication volume. Different colors represent different countries. The size of the arcs in the circular diagram represents the volume of publications, while the connecting lines in the center indicate the collaboration relationships between countries, with the thickness of the lines representing the number of collaborative papers. (**B**) The top 10 countries/regions by total citation count. (**C**) The top 10 countries/regions by average citation count per article.

According to the geographical distribution of the corresponding authors, a total of 54 countries/regions worldwide have conducted research on AI in nursing decision-making. A cluster analysis of countries/regions was performed using VOSviewer (Figure 3A). In the figure, the diameter of the circles represents the number of publications by country/region, while the thickness of the lines indicates the strength of collaboration between countries/regions. The results show that the USA is the most prolific country in this field, with 86 publications, representing 36.13% of the total number of papers. The most frequent collaboration is between the USA and Canada, while the USA also maintains close ties with other countries such as South Korea, Italy, China, the Netherlands, and Saudi Arabia. China is the second most prolific country, with 35 publications, accounting for over 14.71% of the total number of papers, followed by Canada, with 28 publications, accounting for over 11.76% of the total number of papers.

To further evaluate the academic impact of research from different countries, we analyzed the total citation counts and average citation counts. In terms of total citations, the United Kingdom ranks first with 1370 citations, followed by the USA, Canada, and China with 955, 203, and 190 citations, respectively (Figure 3B). In terms of average citations, the United Kingdom also stands out, ranking first with 124.50 citations, followed by Finland with 40.30 citations. Although the USA and China have higher total publication numbers and total citation counts, their average citation counts are relatively low (Figure 3C). In contrast, despite the United Kingdom’s lower total publication output, its total citation count (t = 10.88, *p* < 0.0001, Cohen’s d = 3.62) and average citation count (t = 26.86, *p* < 0.0001, Cohen’s d = 8.95) are significantly higher than those of other countries, indicating that the United Kingdom demonstrates research leadership in terms of research quality and influence.

### 3.3. Contribution of Institutions to Publications

A statistical analysis of the publishing institutions shows that Columbia University is the top university, with 15 papers (Table 1). The University of Toronto and the University of Pennsylvania ranked second and third with 10 and 9 publications, respectively. The number of citations shows that Harvard Medical School ranked first with 236 citations, indicating that its research results have a high impact on the academic community. Columbia University and the University of British Columbia ranked second and third with 223 and 166 citations, respectively. Overall, Columbia University and Harvard Medical School excelled in both the number of publications and citations, demonstrating their strengths in both the quantity and quality of research. Most of the top 10 institutions in terms of publications are from the USA, which indicates that the USA has invested a great deal of attention in the field of “AI in nursing decision making”.

**Figure 4:**
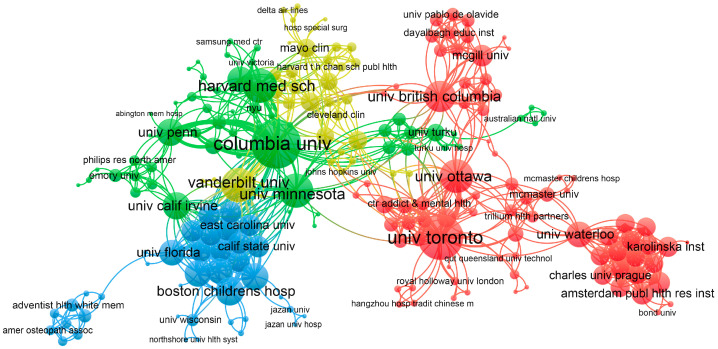
Co-occurrence network of institutions. Different colors represent different clusters. The sizes of the circles indicate the total links, while the lines represent the collaboration relationships between institutions.

To further investigate the collaborations between institutions, we conducted an institutional cluster analysis, where VOSviewer categorized institutional collaborations into four closely related blocks with significant connections and frequent communication between institutions. The collaboration map shows that Columbia University is located in the center of the green cluster and the University of Toronto is located in the center of the red cluster, which are important centers of collaboration between these two institutions (Figure 4).

### 3.4. Contribution of Authors to Publications

**Figure 5:**
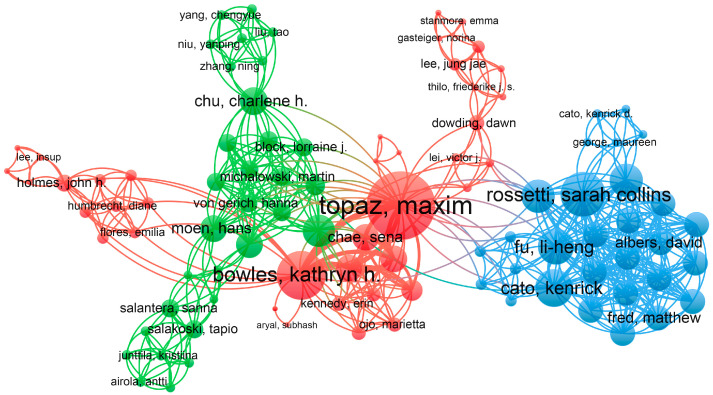
Co-occurrence network of authors. Different colors represent different clusters. The sizes of the circles indicate the total links, while the lines represent the collaboration relationships between authors.

In Table 2, we list the top 10 authors by citation count. Halpern, Yoni, and Horng, Steven, despite having only 2 publications each, rank first with 262 citations, indicating that their work exerts considerable scholarly impact in the academic community. These two papers, respectively, introduce “a method for creating an automated trigger for sepsis clinical decision support at emergency department triage using AI” and “a technique for electronic medical record phenotyping using AI” [26,27]. The four authors ranked second in citations are co-authors of one of these two papers. Topaz, Maxim, ranked third in citations, has published 7 papers with 162 citations and a total link strength of 49. Among these 10 authors, he has the highest number of publications, and his total link strength is significantly higher than others, demonstrating his active research in this field and the extensive connections of his work with other studies. Topaz, Maxim primarily focuses on “AI for automated recognition of nursing notes” and “the role of AI in clinical decision support within home healthcare” [28,29,30,31,32]. Figure 5 illustrates the collaborative relationships among the authors. Clustering information reveals that they can be divided into three clusters. Topaz, Maxim, and Bowles, Kathryn H., who have the highest number of publications, are central figures in the red cluster, highlighting their significant influence in the field of AI in nursing decision making. While authors within each cluster exhibit close collaborative relationships, forming relatively stable research networks, inter-cluster collaboration remains limited. Strengthening academic exchanges and cooperation across clusters is an important direction for future development.

### 3.5. Contribution of Journals to Publications

**Figure 6:**
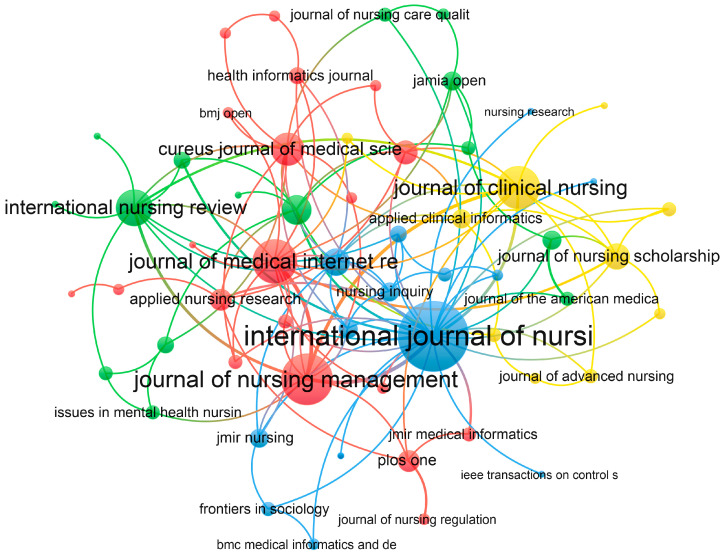
Contribution of journals to publications. These journals were divided into four clusters, represented by red, green, blue, and yellow, respectively. The sizes of the circles and the font size used for journal names indicate the total links, while the lines represent the collaboration relationships between authors.

We conducted a statistical analysis of the journals in which the papers were published, and Table 3 presents the top 10 journals by the number of papers published. *Computers Informatics Nursing* had the highest number of publications (11, 4.62%), followed by the *Journal of Nursing Management* (9, 3.78%). Among these 10 journals, the *International Journal of Nursing Studies* maintained the highest impact factor (IF = 7.5), followed by the *Journal of Medical Internet Research* (IF = 5.8), both of which belong to the Q1 category. Additionally, 50% of these journals are classified as Q1, which, to some extent, reflects that the research achievements in the field of AI in nursing decision making may not yet be fully mature, with room for improvement in academic influence and international recognition. A citation analysis of the journals revealed that they are clustered into four groups based on citation relationships (Figure 6). The *International Journal of Nursing Studies*, *Journal of Nursing Management*, *International Nursing Review*, and *Journal of Clinical Nursing* are the cores of the blue, red, green, and yellow clusters, respectively, indicating their strong connections with other journals.

### 3.6. Keyword Clusters and Evolution

#### 3.6.1. Keyword Clusters Analysis

**Figure 7:**
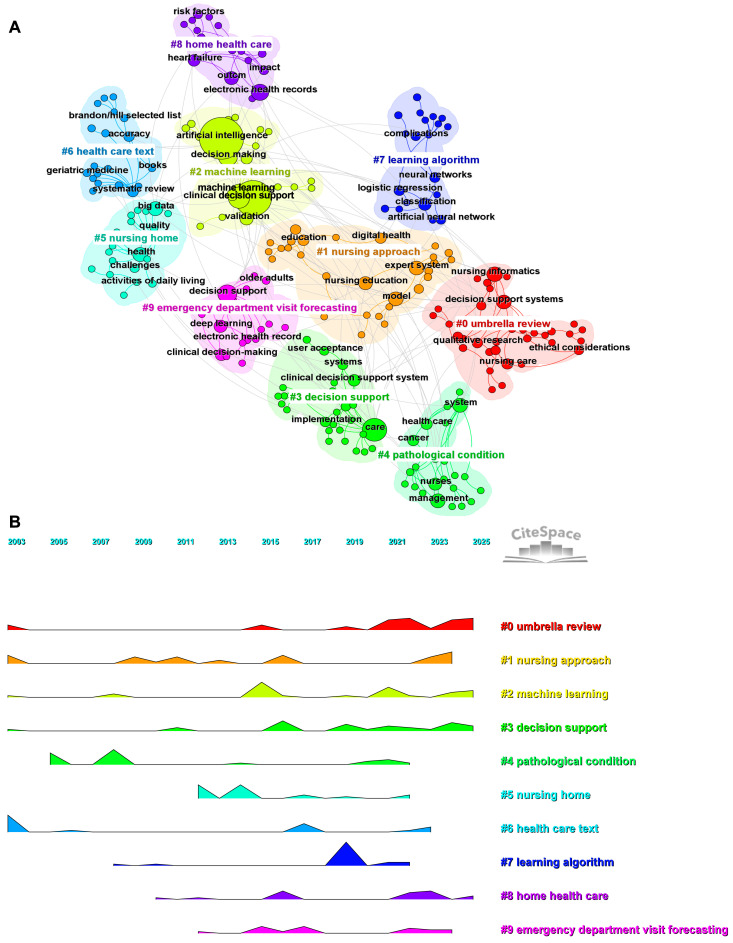
Keyword cluster analysis. (**A**) Keyword cluster network. Different colors represent different clusters, with the top 5 most frequent keywords displayed for each cluster. The text in the white matrix represents the theme of each cluster. (**B**) Keyword cluster time analysis. Each row represents a cluster, with the timeline from 2003 to 2025 depicted from left to right.

Keyword clustering is a method to understand the development trends and frontier research in a particular field. The top 10 keywords by frequency are artificial intelligence (n = 108), machine learning (n = 67), care (n = 31), nursing (n = 28), natural language processing (n = 20), clinical decision support (n = 18), risk (n = 17), mortality (n = 15), electronic health records (n = 13), and model (n = 13). Using CiteSpace, the keywords were clustered into thematic groups, with a modularity Q value of 0.6892 and a weighted mean silhouette S value of 0.9046, indicating a highly stable and reliable clustering structure (Figure 7A). The largest group is “umbrella review” (Cluster #0), followed by “nursing approach” (Cluster #1), “machine learning” (Cluster #2), “decision support” (Cluster #3), “pathological condition” (Cluster #4), “nursing home” (Cluster #5), “health care text” (Cluster #6), “learning algorithm” (Cluster #7), “home health care” (Cluster #8), and “emergency department visit forecasting” (Cluster #9). The clustering results include multiple specific application scenarios, such as “nursing home”, “home health care”, and “emergency department visit forecasting”. This indicates that the application of AI in nursing decision-making is not limited to theoretical research but has also penetrated practical medical and nursing scenarios, particularly in areas such as aging societies and emergency medical care, which have garnered significant attention. A temporal analysis of the clusters reveals that research directions such as “umbrella review” (Cluster #0), “machine learning” (Cluster #2), “decision support” (Cluster #3), and “home health care” (Cluster #8) are still ongoing (Figure 7B).

#### 3.6.2. Keyword Evolution Analysis

**Figure 8:**
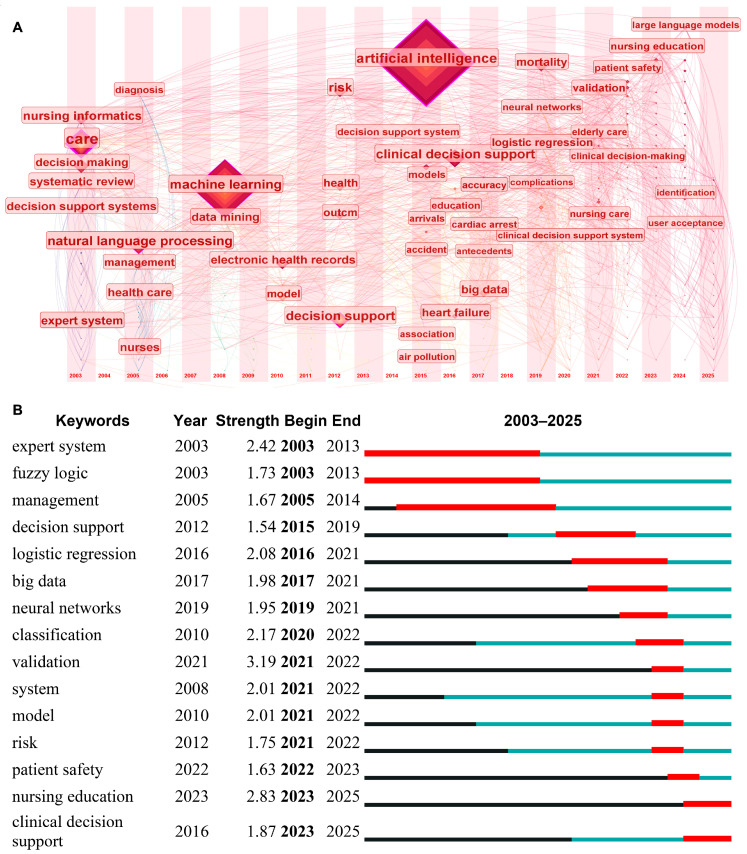
Time flow analysis of keywords. (**A**) Co-occurrence network and time analysis of keywords. The size of the diamond represents the frequency of keyword occurrences. The horizontal axis indicates the time of keyword appearances, covering the time span from 2003 to 2025. (**B**) Top 15 keywords with the highest citation bursts.

A keyword time analysis reveals that, in recent years, keywords such as “patient safety”, “nursing education”, “large language models”, “identification”, and “user acceptance” have begun to emerge (Figure 8A). These new keywords indicate a shift in the research focus of the field from traditional technology-driven approaches to broader application scenarios and societal impacts, such as nursing education, patient safety, and user acceptance. Natural language processing (NLP) technologies, particularly those based on large language models (e.g., ChatGPT), have demonstrated significant potential in nursing decision-making.

A keyword burst analysis shows that the most active keyword in terms of citation bursts is “validation”, with a burst strength of 3.19, and it exhibited a notable citation burst between 2021 and 2022 (Figure 8B). This suggests that the academic community places a high value on the reliability and effectiveness of AI in nursing decision-making applications. Early research hotspots focused on “expert systems” and “fuzzy logic”. In recent years, keywords such as “patient safety”, “clinical decision support”, and “nursing education” have gradually entered a state of burst, indicating that current research hotspots are concentrated on how to enhance patient safety, optimize clinical decision support systems through AI technologies, and improve nursing staff’s understanding and application capabilities of AI technologies through education.

## 4. Discussion

This study conducts a comprehensive bibliometric methods analysis to systematically analyze the current state and development trends of AI in nursing decision-making. Based on 238 publications from the Web of Science Core Collection, we not only quantify the research output in this field but also provide an in-depth exploration of research hotspots and future directions. The study found that the number of AI-related publications in nursing decision-making demonstrated consistent growth. Particularly since 2019, the publication volume has exhibited near-exponential growth, which reflects the increasing significance of AI in nursing decision-making. This trend is primarily driven by advancements in machine learning, natural language processing, and data analytics, enabling more researchers to utilize this technology for in-depth studies.

At the national/regional level, the USA leads in publication volume in this field, contributing 36.13% of the total publications, which is closely linked to its long-term investments in AI and medical technology. China, ranking second in publication volume, has demonstrated rapid growth in the AI field in recent years. However, despite the high total publication volume and citation counts in both the USA and China, their average citations per paper remain relatively low. This disparity may stem from a proliferation of exploratory studies in high-output countries, diluting the average impact. In contrast, the United Kingdom and Finland demonstrate exceptional performance in terms of average citations per paper. Notably, the United Kingdom ranks highest in both total and average citation counts, indicating a strong academic influence and recognition. Although Finland has a lower publication volume, its high-quality research findings are equally noteworthy.

International collaboration has played a crucial role in advancing AI research in nursing decision-making. The USA maintains close collaborations with Canada, South Korea, Italy, China, the Netherlands, and Saudi Arabia, forming an international research network centered around the USA. However, the current collaborative network still has certain limitations. The network is predominantly concentrated in regions such as North America, East Asia, and parts of Europe, while participation from other areas, including Africa, Latin America, and South Asia, remains relatively low. Strengthening cross-regional collaborations, particularly with Europe, Africa, and Latin America, will be beneficial in promoting global knowledge sharing.

The outstanding performance of Columbia University and Harvard Medical School, in terms of publication volume and citation counts, highlights their leading role in innovation within this field. Highly cited authors, such as Halpern, Yoni, and Topaz, Maxim, have significantly contributed to academic discussions on AI applications in nursing. Analysis shows that Halpern, Yoni, and Topaz, Maxim maintain close collaboration, focusing on AI applications in emergency department triage and electronic medical record (EMR) phenotyping. They have co-authored two papers, with one ranking first in citation count at 262. The first paper is a retrospective, observational cohort study, including all consecutive emergency department (ED) patient visits between 17 December 2008 and 17 February 2013 [26]. The study results indicate that, compared to prior research that relied solely on structured data (e.g., vital signs and demographic information), integrating free-text analysis significantly improved the differentiation capability for infection identification. The second paper established a phenotype repository comprising 42 publicly defined phenotypes [27]. This repository is expected to represent all information within EMRs in a fine-grained manner, including both structured and unstructured data, thereby providing a stronger foundation for personalized recommendations and clinical decision support. These institutional and individual contributions signal a shift from theoretical exploration to practical applications, a trend mirrored in the keyword evolution.

Keyword analysis traces this shift from early technologies like “expert systems” and “fuzzy logic” to modern methods like machine learning and NLP. For instance, the development of large language models, such as ChatGPT, has further propelled the application of natural language processing technologies in nursing documentation and decision support [33,34,35]. Saban, Mor et al. utilized a validated framework prompt to generate nursing care plan recommendations, and the results showed that the care plans generated by ChatGPT were highly consistent with the gold standard in terms of scope and nature [33]. However, another study by Saban, Mor et al. compared the diagnostic accuracy and response format of ICU nurses with generative AI models, such as ChatGPT-4 and Claude-2.0 [34]. The results revealed that nurses outperformed AI in diagnostic accuracy in open-ended clinical scenarios, although certain AI models excelled in standardized cases. Similarly, Dubovi, Ilana et al. found that ChatGPT exhibited uncertainty and a tendency toward over-triage in clinical decision-making, and its performance was suboptimal upon re-evaluation [14]. Although AI demonstrated faster response times, the content of its responses often exhibited verbosity. Overall, AI should serve as a complementary tool rather than a replacement and still requires improvement in complex scenarios that demand holistic judgment. Future efforts should focus on enhancing its generalization capabilities in decision-making and optimizing response formats to leverage its strengths, thereby enabling safe and effective support for clinical decision-making.

The evolution and burst analysis of keywords reveals that, in recent years, the emergence of terms such as “patient safety”, “nursing education”, and “user acceptance” indicates a shift in research focus from purely technology-driven approaches to a greater emphasis on the practical impact of AI on nursing systems and professionals. For instance, Aoki, Naomi, explored the initial trust of the public in the application of AI in public services, highlighting concerns about AI fully taking over nursing plans and underscoring the importance of maintaining human involvement in AI applications [36]. Hariri, Fatemeh, and colleagues investigated the ethical issues surrounding the use of AI in healthcare, particularly in nursing, revealing gaps in research on data ownership rights and AI ethics, and proposing new cases to address these gaps [37]. Additionally, through a case study of the SENSOMATT project, the research delved into GDPR and privacy issues, providing valuable insights for stakeholders in navigating data protection, ethics, and regulatory compliance in AI-driven healthcare.

Our bibliometric study has several limitations. First, this research only includes the literature in English, overlooking significant publications in other languages. Second, specialized medical databases such as PubMed and CINAHL contain an extensive literature on nursing practice and clinical decision-making. However, due to compatibility issues between the current bibliometric analysis tools (CiteSpace/VOSviewer) and CINAHL’s data format, the retrieved results could not be incorporated into the analysis. This study exclusively employed Web of Science as the literature retrieval database. Although its Core Collection (SCI/SSCI) covers multidisciplinary fields and maintains rigorous journal selection criteria, sole reliance on Web of Science may have led to the omission of certain nursing-related studies, thereby introducing limitations. Future research should optimize search strategies by incorporating multi-source data and mixed methods to more comprehensively reflect developments in this field. Third, high-quality papers published recently may not receive adequate attention due to their short publication time and low citation frequency. Fourth, while this bibliometric analysis reveals key research trends in AI in nursing decision-making, bibliometric indicators (e.g., citation counts and co-occurrence networks) primarily reflect research activity and influence, rather than serving as direct measures of research quality or clinical impact. Lastly, although this study employed a rigorous triple-concept screening strategy (AI × nursing × decision-making terms) using Web of Science’s Topic Search field covering titles, abstracts, and keywords, the title/abstract-based screening may have led to the inclusion of some studies where AI applications only peripherally addressed nursing decision-making. Additionally, certain critical information might be exclusively detailed in the full text, making it challenging to comprehensively assess study relevance through abstract screening alone. These factors could potentially impact the accuracy of the final included literature. Future research could benefit from full-text screening to further validate the findings.

## 5. Conclusions

A systematic bibliometric analysis of 238 publications reveals that research on AI in nursing decision-making is experiencing sustained and accelerated growth, with the United States, China, and Canada emerging as core contributors. Columbia University and the journal *Cin-Computers Informatics Nursing* emerge as the leading institution and platform, respectively. NLP technologies demonstrate significant potential in this field, with research hotspots focusing on application scenarios such as “nursing homes”, “home healthcare”, and “emergency department visit prediction”. Notably, ”patient safety” and “user acceptance” have emerged as critical emerging themes. This study delineates the current landscape and impact of AI in nursing decision-making while providing pivotal insights to guide future research directions.

## Figures and Tables

**Table 1:** Contribution of institutions to publications.

Organization	Documents	Citations	Total Link Strength
Columbia University	15	223	59
University of Toronto	10	142	53
University of Pennsylvania	9	86	26
Harvard Medical School	8	236	36
Mayo Clinic College of Medicine and Science	7	97	15
University of Minnesota Twin Cities	6	120	36
Vanderbilt University	5	33	36
University of Ottawa	5	4	35
University of British Columbia	5	166	31
Brigham and Women’s Hospital	5	58	24

**Table 2:** Contribution of authors to publications.

Author	Documents	Citations	Total Link Strength
Halpern, Yoni	2	262	8
Horng, Steven	2	262	8
Jernite, Yacine	1	174	5
Nathanson, Larry A.	1	174	5
Shapiro, Nathan I.	1	174	5
Sontag, David A.	1	174	5
Topaz, Maxim	7	162	49
Chu, Charlene H.	2	122	20
Moen, Hans	2	105	19
Peltonen, Laura-Maria	2	105	19

**Table 3:** Contribution of journals to publications.

Sources	Papers	IF	JCR
*Cin-Computers Informatics Nursing*	11	1.3	Q3
*Journal of Nursing Management*	9	3.7	Q1
*Applied Clinical Informatics*	6	2.1	Q4
*Journal of Medical Internet Research*	6	5.8	Q1
*Journal of The American Medical Informatics Association*	6	4.7	Q1
*Cureus Journal of Medical Science*	5	1	Q3
*International Journal of Medical Informatics*	5	3.7	Q1
*International Journal of Nursing Studies*	5	7.5	Q1
*Jmir Research Protocols*	5	1.4	Q3
*Journal of Biomedical Informatics*	5	4	Q2

## Data Availability

Not applicable.

## Public Involvement Statement

No public involvement in any aspect of this research.

## Guidelines and Standards Statement

Despite the widespread use of bibliometric indicators in scientific analysis, there are no agreed-upon guidelines or standards for reporting bibliometric studies.
